# Supplementary material for: Single pulse all-optical toggle switching of magnetization without gadolinium in the ferrimagnet Mn2RuxGa
Source: Nat Commun. 2020 Sep 7;11:4444. doi: 10.1038/s41467-020-18340-9 (PMC7477543; doi:10.1038/s41467-020-18340-9)
Supplement: Supplementary file 1 — Supplementary Information [file 41467_2020_18340_MOESM1_ESM.pdf]

Supplementary Information

Single pulse all-optical toggle switching of magnetization without gadolinium in the ferrimagnet Mn_2Ru_xGa

Banerjee *et al.*

Supplementary Note 1

STRUCTURAL AND MAGNETIC CHARACTERIZATION OF $Mn_2Ru_{1.0}Ga$

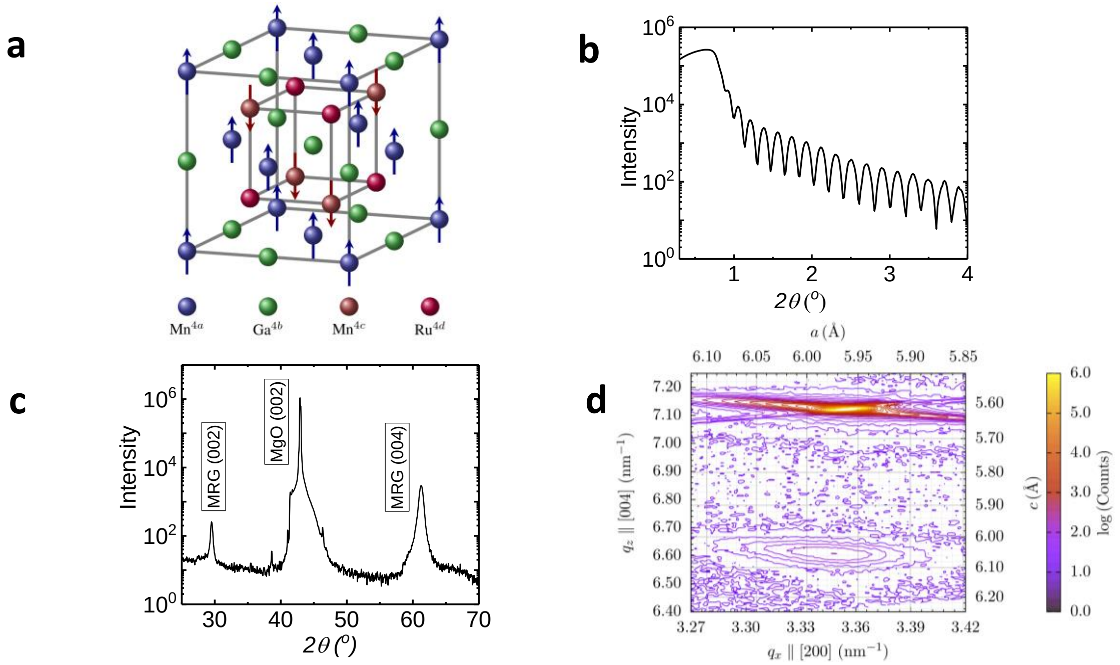

Supplementary Figure 1: (a) Diagram of the inverted Heusler (XA) crystal unit cell of typical MRG film. Characterisation of $Mn_2Ru_{1.0}Ga$ (MRG): (b) X ray reflectivity pattern of the MRG thin film. Fitting gives a thickness of 42.8 nm and a density of 8.2 g. cm^{-3}, (c) X ray diffraction pattern of MRG thin film on MgO (001) substrate. (d) Reciprocal space map of MgO (113) peak and MRG (204) peak with lattice parameters calculated with respect to the MRG unit cell.

MRG crystallises in an inverted Heusler (XA) structure of space group $F\bar{4}3m$ with two crystallographically inequivalent magnetic Mn atoms at Wyckoff positions 4*a* and 4*c* and Ga and Ru atoms occupy the 4*b* and 4*d* positions, respectively, as shown in Suppl. Fig. 1a. Note the Mn(4*c*) sublattice is non-centrosymmetric. Due to the biaxial non-volume conserving strain of the MgO substrate the unit cell of MRG is tetragonally distorted and hence the space group is reduced to $I\bar{4}2m$. X-ray data on the $Mn_2Ru_{1.0}Ga$ film are shown in Suppl. Fig. 1b, c and d. The X-ray reflectivity (XRR) pattern shown in Suppl. Fig. 1b has been fitted using X'Pert Reflectivity software and the film thickness was calculated to be 42.8 nm. The X-ray diffraction (XRD) pattern in Suppl. Fig. 1c exhibits (002) and (004) reflections from the MRG, together with peaks from the MgO substrate. The c-parameter calculated from the (004) reflection is 604.7 pm. A reciprocal space map (RSM) of the MRG film in Suppl. Fig. 1d confirms the c-parameter obtained from XRD, and shows a distribution of a-parameters around the central

value of 595.8 pm, which corresponds to that of MgO. This demonstrates how substrate strain induces a ~ 1% tetragonal elongation of the MRG unit cell since the c/a ratio is 1.01, giving rise to the perpendicular magnetic anisotropy found in all MRG films.

Supplementary Note 2

DEPENDENCE OF MAGNETIZATION ON APPLIED MAGNETIC FIELD FOR $Mn_2Ru_{0.65}Ga$ AND $Mn_2Ru_{1.0}Ga$

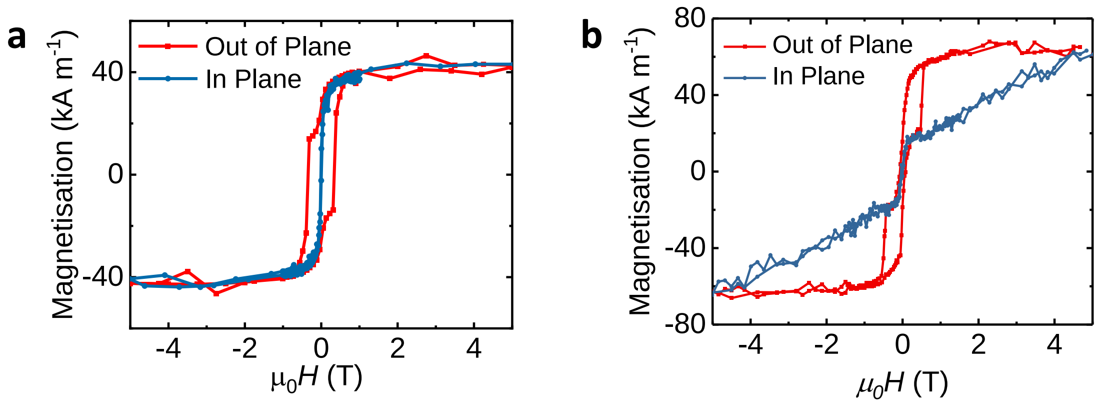

Supplementary Figure 2: Magnetization versus field applied in plane and out of plane at 300 K (a) for the $Mn_2Ru_{0.65}Ga$ film and (b) for the $Mn_2Ru_{1.0}Ga$ film.

The dependence of magnetization on the field applied out of plane and in plane at 300 K relative to the surface of the thin film was measured for $Mn_2Ru_{0.65}Ga$ and $Mn_2Ru_{1.0}Ga$ films (See Suppl. Fig. 2) using SQUID magnetometry, which have T_{comp} below and above RT respectively. From the curves, the saturation magnetizations are 40 and 65 kA . m^{-1}, respectively. Interestingly, we observe a soft component in the out-of-plane, as well as the in-plane magnetization, which originates partly from the non-collinearity of the two exchange-coupled antiferromagnetically aligned Mn magnetic sublattices.

Supplementary Note 3

MEASUREMENT OF OPTICAL AND TRANSPORT MAGNETOMETRY OF $Mn_2Ru_{1.0}Ga$

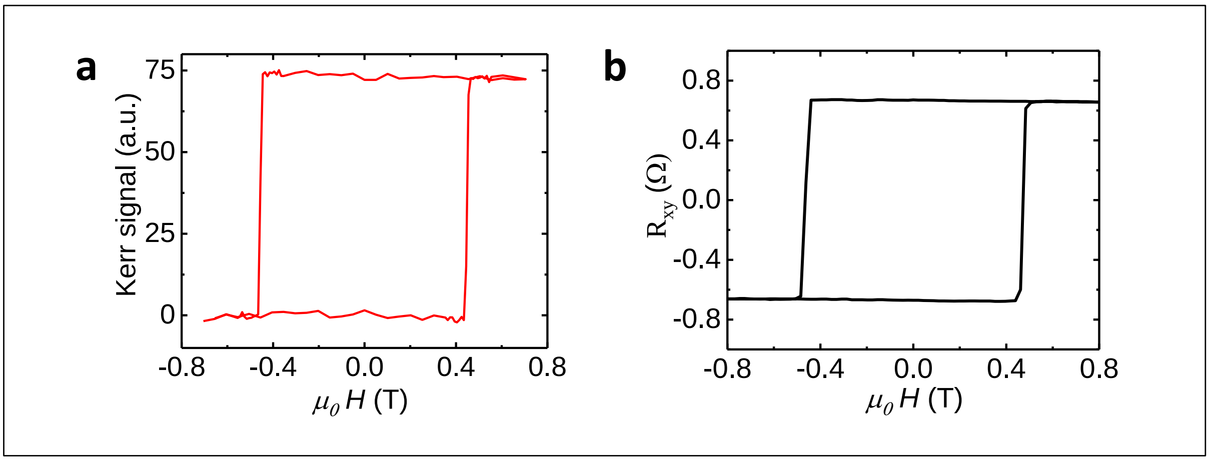

Supplementary Figure 3: Measured hysteresis loops of $Mn_2Ru_{1.0}Ga$ via (a) Kerr microscopy and (b) anomalous Hall effect.

We have measured the magnetic hysteresis loops using polar Kerr effect (λ = 630 nm) and anomalous Hall effect in the perpendicular $Mn_2Ru_{1.0}Ga$ film, which are compared in Suppl. Fig. 3. In contrast to the two-step switching observed in the SQUID loop (See Suppl. Fig. 2b), here the square hysteresis loops exhibit straightforward single-step switching, with an average switching field of 460 mT in both cases. Whereas the SQUID probes the net magnetic moment, the difference of the nearly-equal sublattice contributions, the optical and electrical transport, on the other hand, relies on the distribution of spin-polarized electrons at or near the Fermi energy, which in MRG reflects the 4*c* sublattice magnetization. Consequently, the hysteresis shown in Suppl. Fig. 3 as well as the domain images presented here reflects the local magnetization state of the 4*c* sublattice. It is therefore possible to explore the local magnetization state even at compensation.

Supplementary Note 4

DETERMINATION OF T_{comp} FOR $Mn_2Ru_{1.0}Ga$

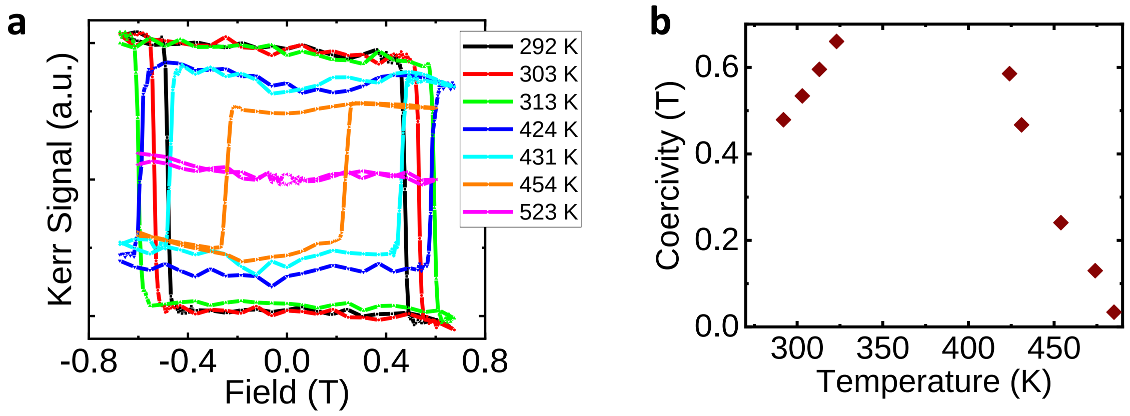

Supplementary Figure 4: Optically measured hysteresis loops for $Mn_2Ru_{1.0}Ga$ at different temperatures (a) and the corresponding variation of coercive field (b).

Supplementary Figure 4a presents the hysteresis loops measured by polar Kerr effect in $Mn_2Ru_{1.0}Ga$ at different temperatures. On approaching T_{comp}, the net moment falls, which increases the anisotropy field and coercivity, until they diverge at T_{comp}. In addition, as the optical measurements probe the Mn(4*c*) sublattice only, a change in the sign of the hysteresis loop is seen upon crossing T_{comp}. The variation of the coercive field as a function of temperature is shown in Suppl. Fig. 4b, from which T_{comp} for this sample is estimated to be ~ 390 K.

Supplementary Note 5

RESULT OF PULSE ENERGY DEPENDENT MEASUREMENT IN MRG SAMPLE WHERE AOS WAS NOT OBSERVED

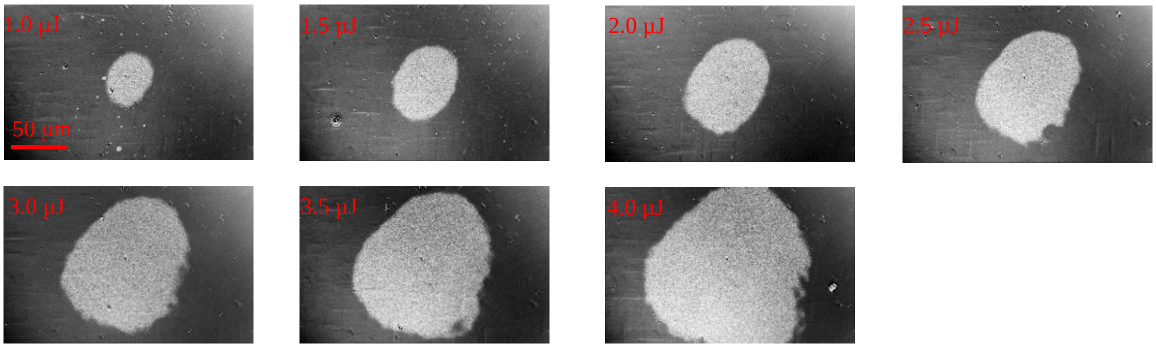

Supplementary Figure 5: Typical Kerr microscope images of MRG after single laser pulse of various pulse energies were irradiated on different positions of the surface. The results show a multidomain pattern has formed.

In the main text, single-pulse all-optical switching (SP-AOS) is presented for MRG samples having T_{comp} above room temperature. No toggling was observed for the MRG samples having T_{comp} below room temperature. Supplementary Figure 5 shows typical Kerr micrographs after the pulse, for different pulse energies. The images reveal the presence of multidomain state with an onset at ~ 1 µJ. This is different to the $Mn_2Ru_{1.0}Ga$ results, where a switched ring was observed around the thermally demagnetized region (See Fig 1c in main text).

Supplementary Note 6

DETERMINATION OF THE SPOT SIZE AND THRESHOLD FLUENCE FOR SWITCHING

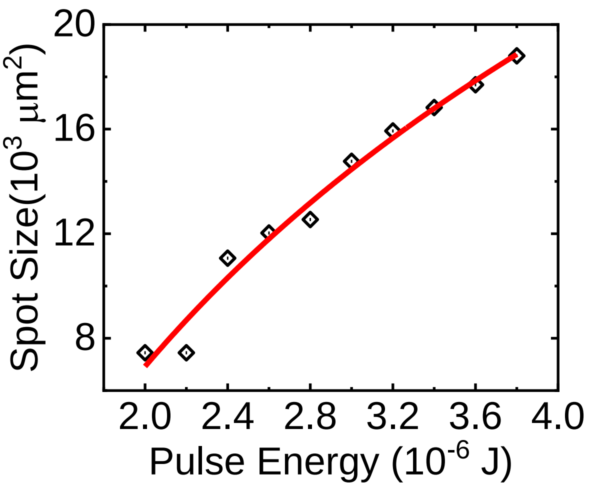

Supplementary Figure 6: Switched domain size as a function of pulse energy for $Mn_2Ru_{1.0}Ga$. The red solid line is a fit to Eqn. 1.

As mentioned in the main text, we have employed the growth of the switched domain size with increasing pulse energy for $Mn_2Ru_{1.0}Ga$ to calculate the laser spot size as well as the threshold fluence for switching using the Liu method[1]. This method exploits the fact that at the edge of the magnetic contrast, the excitation fluence is equal to the threshold fluence. By assuming a Gaussian pulse-shape, the switched area can be determined as,

$$A_S = A_0 \ln\left(\frac{E}{E_{\mathrm{th}}}\right) \qquad \text{.........(1)}$$

where A_S is the switched area corresponding to pulse energy E, A_0 is the laser spot area and E_{th} is the threshold pulse energy. Supplementary Figure 6 presents the variation of the switched area as a function of pulse energy, from which the spot area and the threshold pulse energy are extracted by fitting with Eqn. 1 to be 18600 μm^2 and 1.4 μJ respectively. The threshold fluence is then calculated by dividing the threshold pulse energy with the laser-spot area, which yields ~7.5 mJ cm^{-2}.

Supplementary Note 7

EVOLUTION OF MAGNETIZATION DYNAMICS WITH INCREASING FLUENCE ABOVE F_{th}

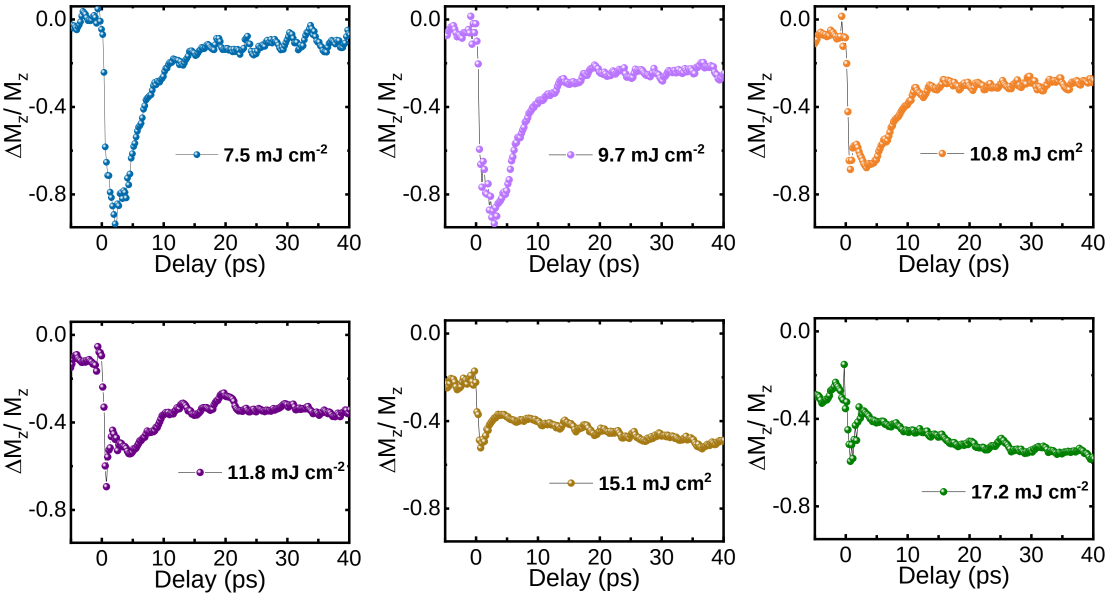

Supplementary Figure 7: Time dependent traces of the ultrafast magnetization dynamics of $Mn_2Ru_{1.0}Ga$ for different laser fluences.

In Suppl. Fig. 7 we have shown the evolution of the ultrafast magnetization dynamics with increasing laser fluence starting from F_{th} (7.5 mJ cm^{-2}). It can be seen that the slope at~ 2 ps changes gradually from positive to negative. In addition, after the initial demagnetization, a partial recovery of the signal is observed, which is a signature of the transfer of angular momentum between the sublattices. These features gradually evolve in the signal after the laser fluence crosses F_{th}, and becomes prominent at ~ 14 mJ cm^{-2}, where the entire spot area is switched (See Fig. 1 in main text).

Supplementary Note 8

SP-AOS MEASUREMENT ON $Mn_2Ru_{0.92}Ga$ USING LASER PULSES OF WAVELENGTH 400 NM

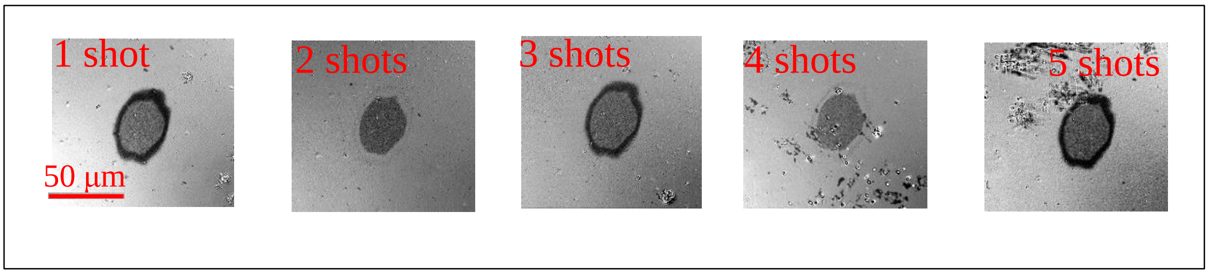

Supplementary Figure 8: Single pulse all optical switching measurement performed on $Mn_2Ru_{0.92}Ga$ using laser pulses of wavelength 400 nm and pulse energy 0.9 µJ. The number of laser pulses the region is exposed to is labelled in each image.

In order to substantiate the thermal origin of SP-AOS in MRG, we examined the response of its magnetization to the laser pulses of different wavelength and polarization. In Suppl. Fig. 8 the response of $Mn_2Ru_{0.92}Ga$ is shown for laser beam of wavelength 400 nm and up to five laser pulses. The results are in line with the observation with 800 nm (See Fig. 2 in main text). Essentially, a decrease in the excitation wavelength decreases the laser spot size, thereby increasing the thermal gradient across it. This directly affects the magnetization profile of the irradiated region and the switching is observed in a relatively narrow ring around the thermally demagnetized area, as compared to the 800 nm case.

Supplementary Note 9

CALCULATION OF TRANSIENT ELECTRON, LATTICE AND SPIN TEMPERATURES USING FOUR TEMPERATURE MODELLING

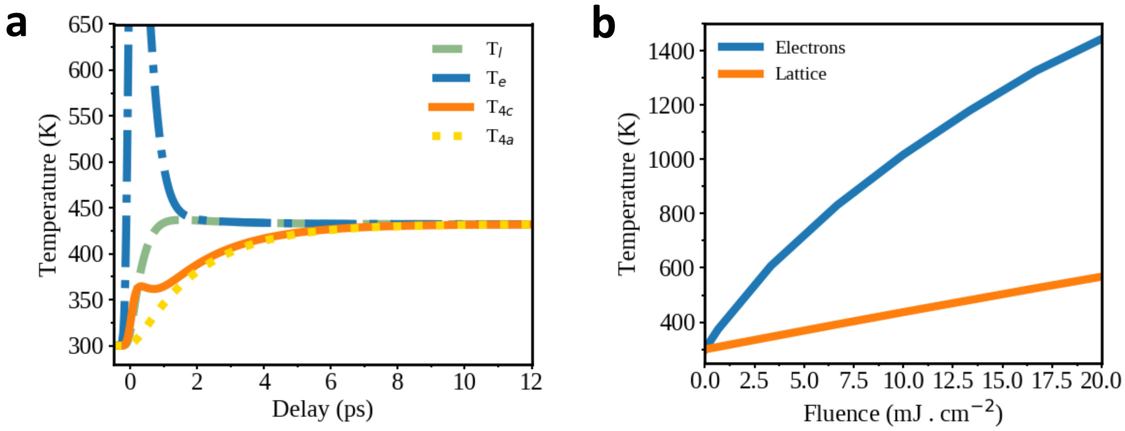

Supplementary Figure 9: (a) Transient temperature variation of electron, lattice, Mn(4*c*) and Mn(4*a*) sublattices calculated using four temperature modelling. The laser fluence is 10.5 mJ.cm^{-2}. (b) Maximum electron and lattice temperature calculated using 4T model for different laser fluences.

Using four temperature modelling, we have calculated the transient rise in the electron, lattice and the spin temperatures for the two sublattices, as shown in Suppl. Fig. 9a and b. The parameters are taken from Ref. 27 in the main text. We assumed a 15% absorption of the laser fluence so as to transiently reach the Curie temperature (550 K) at the threshold fluence for thermal demagnetization. The resulting increase in the lattice temperature corresponding to F_{th} comes out to be equivalent to reaching T_{comp} (390 K for the sample discussed in the main text).

Supplementary Note 10

COMPARISON OF TEMPERATURE DEPENDENCE OF MAGNETIZATION IN MRG AND GdFeCo

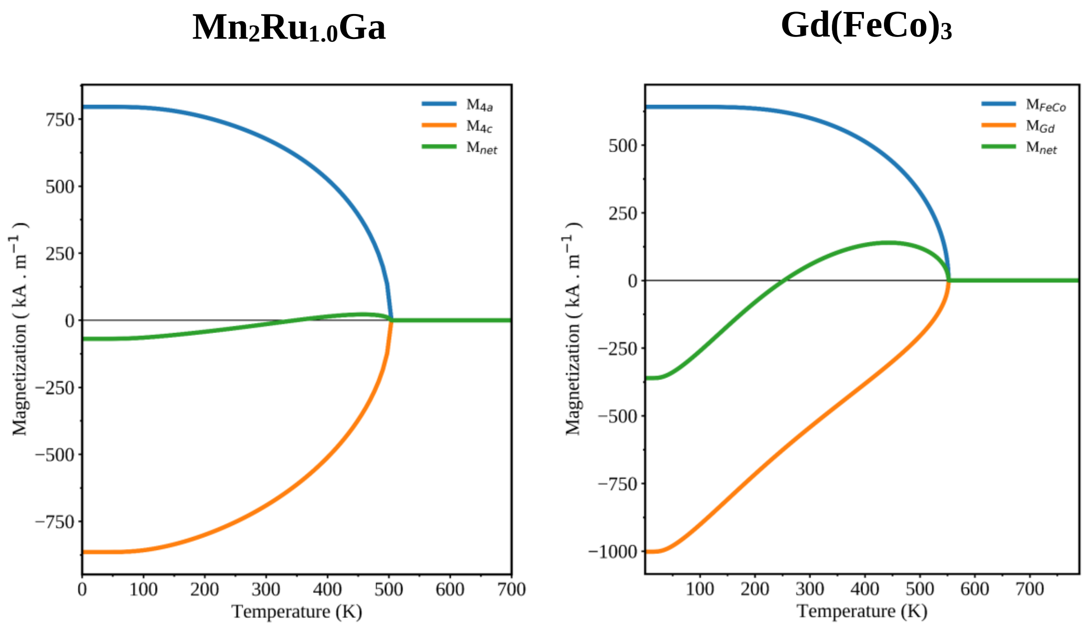

Supplementary Figure 10: Representation of the sublatttice magnetizations with respect to temperature calculated in the mean field approach for $Mn_2Ru_{1.0}Ga$ and $Gd(FeCo)_3$.

Supplementary Table 1

Outcome of single-pulse excitation in various MRG samples. In some cases, where similar behaviour was found for films with similar composition, only one entry is shown. Values of T_{comp} shown in black are experimentally measured, while the others (shown in blue) are interpolated.

**Sample**	**T_{comp} (K)**	**Coercive Field (mT)**	**Switching Observed?**
$Mn_2Ru_{0.5}Ga$	75	150	No
$Mn_2Ru_{0.55}Ga$	80	170	No
$Mn_2Ru_{0.60}Ga$	130	260	No
$Mn_2Ru_{0.62}Ga$	145	350	No
$Mn_2Ru_{0.63}Ga$	160	370	No
$Mn_2Ru_{0.65}Ga$	165	440	No
$Mn_2Ru_{0.7}Ga$	245	740	No
$Mn_2Ru_{0.9}Ga$	310	> 1000	Yes
$Mn_2Ru_{0.92}Ga$	315	> 1000	Yes
$Mn_2Ru_{0.93}Ga$	320	> 1000	Yes
$Mn_2Ru_{0.94}Ga$	325	> 1000	Yes
$Mn_2Ru_{0.95}Ga$	375	600	Yes
$Mn_2Ru_{1.0}Ga$	390	480	Yes

Supplementary Table 2

MAGNETIC PROPERTIES OF Mn_2Ru_xGa AND $Gd(FeCo)_3$

Magnetic properties of Mn_2Ru_xGa and $Gd(FeCo)_3$ (i.e. GdFeCo). T_c and T_{comp} are respectively the Curie and compensation temperatures. M_i is the magnetization of the sublattice i and M_{net} is the net magnetization of the system. τ_i are the characteristic demagnetization times for sublattices i = 4a, 4c in MRG and i = Gd, FeCo in $Gd(FeCo)_3$. $\tau_{e\text{-}l}$ is the characteristic time associated to the energy transfer between the hot electronic system and the lattice.

	Mn_2Ru_xGa	$Gd(FeCo)_3$
**Structure**	Cubic Heusler XA	Amorphous
**T_c (K)**	500* (Mn_2RuGa)	500[5] ($Gd_{22}Fe_{9.8}Co_{68.2}$)
**T_{comp} (K)**	390*,† ($Mn_2Ru_{1.0}Ga$)	250[4] ($Gd_{25}Fe_{65.6}Co_{9.4}$)
**$M_{4a/FeCo}$ (kA . m^{-1})**	790* ($Mn_2Ru_{1.0}Ga$)<br>550[2] ($Mn_2Ru_{0.61}Ga$)	640‡ ($Gd_{25}Fe_{65}Co_{10}$)
**$M_{4c/Gd}$ (kA . m^{-1})**	860* ($Mn_2Ru_{1.0}Ga$)<br>590[2] ($Mn_2Ru_{0.61}Ga$)	1000‡ ($Gd_{25}Fe_{65}Co_{10}$)
**M_{net} (kA . m^{-1})**	70† (0 K) ($Mn_2Ru_{1.0}Ga$)<br>40[2] (0 K)($Mn_2Ru_{0.61}Ga$)	360‡ (0 K) ($Gd_{25}Fe_{65}Co_{10}$)
**$\tau_{4a/Gd}$ (ps)**	8.0[3] ($Mn_2Ru_{0.7}Ga$)	0.43[4] ($Gd_{25}Fe_{65.6}Co_{9.4}$)
**$\tau_{4c/ FeCo}$ (ps)**	0.5 ($Mn_2Ru_{1.0}Ga$),<br>0.5/8.0[3] ($Mn_2Ru_{0.7}Ga$)	0.1[4] ($Gd_{25}Fe_{65.6}Co_{9.4}$)<br>4.0/150[5] ($Gd_{22}Fe_{9.8}Co_{68.2}$)
**$\tau_{e\text{-}l}$ (ps)**	2.0[3] ($Mn_2Ru_{0.7}Ga$)	1.5[4] ($Gd_{25}Fe_{65.6}Co_{9.4}$)<br>5.0[5] ($Gd_{22}Fe_{9.8}Co_{68.2}$)

* From static MOKE vs Temperature measurements.

† From SQUID/XRD measurements.

‡ Estimated for atomic spins μ_{Gd} =7.6 μ_B and $\mu_{Fe/Co}$ =1.6 μ_B.

Supplementary References

1. Liu, J. M. Opt. Lett. **7**, 196 (1982).
2. Fowley *et al.* Phys. Rev. B **98**, 220406(R) (2018).
3. Bonfiglio *et al.* Preprint at https://arxiv.org/abs/2003.01420 (2020).
4. Radu *et al.* Nature **472**, 205-209 (2011).
5. Mekonnen *et al.* Phys. Rev. B **87**, 180406(R) (2013).
